# Cholesterol-Sensitive Cdc42 Activation Regulates Actin Polymerization for Endocytosis via the GEEC Pathway

**DOI:** 10.1111/j.1600-0854.2007.00565.x

**Published:** 2007-04-25

**Authors:** Rahul Chadda, Mark T. Howes, Sarah J. Plowman, John F. Hancock, Robert G. Parton, Satyajit Mayor

**Affiliations:** 1 National Centre for Biological Sciences, UAS-GKVK Campus, Bellary Road, Bangalore 560065, India; 2 Institute for Molecular Bioscience, University of Queensland, Queensland 4072, Australia; 3 Centre for Microscopy and Microanalysis, University of Queensland, Queensland 4072, Australia

**Keywords:** actin, Cdc42, cholesterol, endocytosis, GEECs, GPI-APs, TIRF

## Abstract

Glycosyl-phosphatidylinositol (GPI)-anchored proteins (GPI-APs) are present at the surface of living cells in cholesterol dependent nanoscale clusters. These clusters appear to act as sorting signals for the selective endocytosis of GPI-APs via a Cdc42-regulated, dynamin and clathrin-independent pinocytic pathway called the GPI-AP-enriched early endosomal compartments (GEECs) pathway. Here we show that endocytosis via the GEECs pathway is inhibited by mild depletion of cholesterol, perturbation of actin polymerization or overexpression of the Cdc42/Rac-interactive-binding (CRIB) motif of neural Wiskott–Aldrich syndrome protein (N-WASP). Consistent with the involvement of Cdc42-based actin nanomachinery, nascent endocytic vesicles containing cargo for the GEEC pathway co-localize with fluorescent protein-tagged isoforms of Cdc42, CRIB domain, N-WASP and actin; high-resolution electron microscopy on plasma membrane sheets reveals Cdc42-labelled regions rich in green fluorescent protein–GPI. Using total internal reflection fluorescence microscopy at the single-molecule scale, we find that mild cholesterol depletion alters the dynamics of actin polymerization at the cell surface by inhibiting Cdc42 activation and consequently its stabilization at the cell surface. These results suggest that endocytosis into GEECs occurs through a cholesterol-sensitive, Cdc42-based recruitment of the actin polymerization machinery.

At the cell surface, there are multiple pathways available for the internalization of membrane proteins and lipids (1–4). Dynamin activity is required for generation of both clathrin/caveolin-coated as well as non-coated endocytic carriers (5–9). In this medley of dynamin-dependent pathways, there is a growing number of dynamin-independent endocytic routes for various cell-surface proteins, including lipid-tethered proteins, such as glycosyl-phosphatidylinositol (GPI)-anchored proteins (GPI-APs) and transmembrane proteins, such as major histocompatibility complex I and Tac, the α-subunit of interleukin-2 receptor (1,10–14). These pathways are clathrin and caveolin independent, and are regulated by different small molecule GTPases. Donaldson and co-workers (13) have suggested that the GTPase Arf6 is associated with one such pathway of non-specific membrane component internalization.

Recent work from our laboratory and others have suggested that the Rho family GTPase Cdc42 is responsible for uptake of specific membrane components such as GPI-APs via a distinct clathrin-independent endocytic pathway that does not require Arf6 function (12,15–17). We have shown that GPI-APs and cholera toxin bound to ganglioside GM1 are internalized by a dynamin-independent mechanism. This is mediated by primary pinocytic carrier vesicles devoid of both clathrin and caveolin, termed CLICs (16), which then fuse to form distinct endosomal compartments termed GPI-AP-enriched early endosomal compartments (GEECs) (12) in a variety of cell types (12,16,18). In fact, recent studies suggest that the GEEC pathway may constitute a specialized, high-capacity endocytic pathway for uptake of specific lipid-anchored proteins (GPI-APs), lipid-binding toxins (16) and bulk-phase fluid (16,17).

At the surface of cells, a fraction of GPI-APs is organized into nanoscale clusters (nanoclusters) that are sensitive to removal of significant amounts of cholesterol from the cell (19,20). Association with these clusters appears necessary for targeting GPI-APs into the GEEC pathway (19), because cross-linking GPI-APs dissociates these proteins from pre-existing clusters, and prevents their endocytosis via GEEC pathway. These observations strongly suggest that the organization of GPI-APs into nanoclusters serves as a sorting signal for specific endocytic routing. Consistent with this hypothesis, exchanging the GPI anchor with a trans-membrane anchor diverts the protein away from the GEEC pathway (12). These results led us to propose that nanoclusters may be induced to form large-scale lipid domains that define a zone of endocytic activity (21). These organized membrane domains can enter cells via tubular invaginations and form CLICs (12,16). However, the endocytic machinery associated with this pathway remains uncharacterized.

In this study, we show that mild depletion of cholesterol from cells inhibits endocytosis via the GEEC pathway in a specific manner. It does not disperse nanoclusters (19), but reduces the number of endocytic events. Consistent with a role for Cdc42-based signalling leading to actin polymerization in the generation of endosomal invaginations, the GEEC pathway is blocked by modifiers of the actin polymerization machinery, latrunculin A (Lat A), cytochalasin D (Cyto D), jasplakinolide (Jas), and overexpression of the Cdc42/Rac-interactive-binding (CRIB) motif of the neural Wiskott–Aldrich syndrome protein (N-WASP). Using total internal reflection fluorescence (TIRF) microscopy and electron microscopy (EM), we show co-localization of Cdc42 with endocytosed fluid phase in nascent endosomes and with GPI-APs at or near the cell surface. Finally, we show by real time TIRF microscopy on live cells that recruitment of single molecules of Cdc42 to the plasma membrane and the generation of actin assembly sites are both perturbed by mild depletion of cholesterol.

## Results

### Cholesterol depletion inhibits endocytosis via the GEEC pathway

It has been shown that endocytic retention of GPI-APs in recycling endosomes is sensitive to levels of cholesterol in cells (22). Here we have examined if the initial steps of endocytosis could be sensitive to lowering of cholesterol levels. Because a major fraction of the fluid phase is endocytosed via this pathway, we also looked at the extent of perturbation of the fluid-phase uptake. Endocytosis of GPI-APs, the fluid phase and transferrin receptor (TfR) was measured in cholesterol-depleted cells, by monitoring the uptake of a fluorescently tagged model GPI-AP [GPI-anchored folate receptor (FR-GPI), bound to fluorescent folate analogs], fluorescently labelled 10-kD dextrans and transferrin (Tf), respectively.

**Figure 1:**
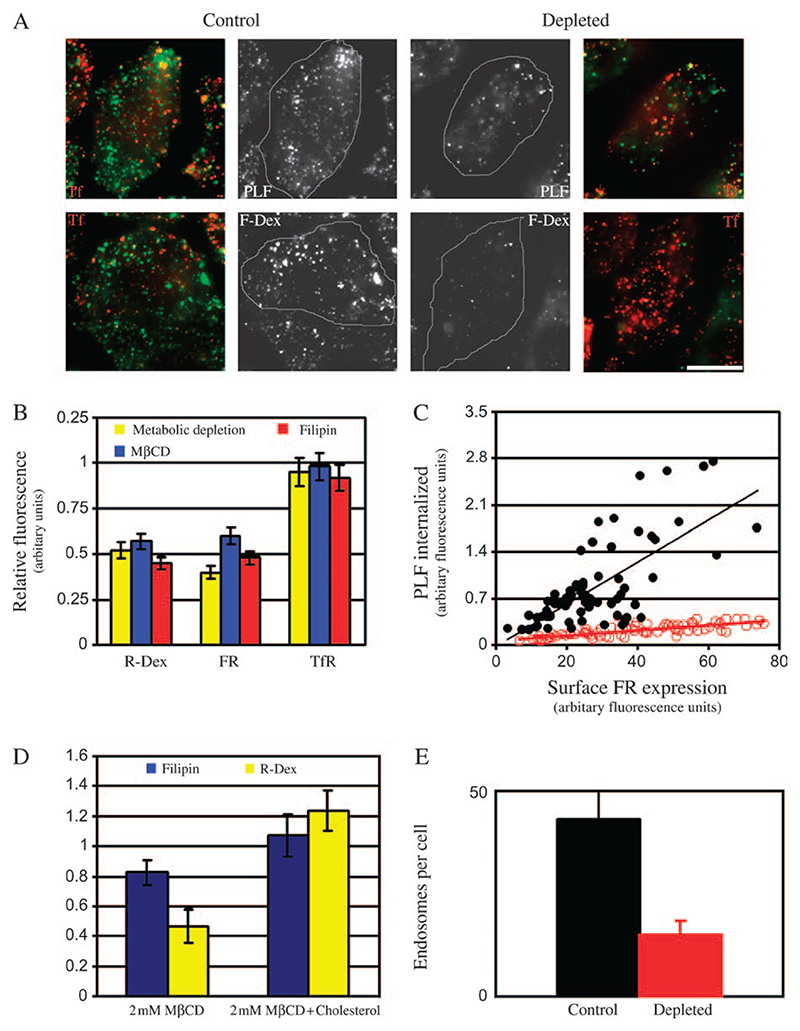
Depletion of cholesterol from cells blocks endocytosis via the GEEC pathway of endocytosis. A) Control (left panels) or cholesterol-depleted (metabolic treatment; right panels) FR-GPI-expressing (FRαTb) CHO cells were incubated for 2 min at 37°C with either A568-Tf and PLF (top panel) or A568-Tf and F-Dex (green) (lower panel), washed, fixed and imaged on high-resolution wide-field microscope. Grayscale images of PLF/F-Dex (green) and A568-Tf-labelled TfR (red)-containing structures were pseudocoloured and colour merged. Scale bar, 10 μm. B) Histogram shows the extent of uptake (5 min) in cells depleted of cholesterol relative to control cells (set to 1), for indicated endocytic tracers [R-Dex, PLF (FR-GPI) and A568-Tf (TfR)]. Cholesterol was depleted via metabolic depletion (yellow bar), of treatment with MβCD (blue) or filipin (red). Values are wt. mean ± SEM obtained across three to five experiments each. C) Scatter plot shows uptake of FR-GPI determined by PLF fluorescence, after a 5-min pulse in control (filled circles) and metabolically depleted cells (open circles) plotted against the respective surface expression of FR-GPI (as marked by PLR). Similar data were obtained from three independent experiments. D) Histogram shows the extent of filipin staining and fluid uptake in cholesterol-depleted and cholesterol-replenished cells, expressed relative to control cells (value of controls set to 1). Cells were treated with 2 mM MβCD for 30 min. Cholesterol was replenished in depleted cells by incubating them with 2 mM MβCD–cholesterol complexes. Control, depleted and replenished cells were stained with filipin to ascertain changes in cholesterol level. Error bars represent wt. mean ± SEM. E) Histogram shows the number of GEECs as marked by PLF and TMR-dextran uptake after a 2-min pulse in control (black bar) and cholesterol-depleted cells (red bar). Bars represented are wt. mean ± SEM obtained across two experiments.

To reduce cholesterol levels by metabolic depletion, folate receptor (FR-GPI)-expressing CHO cells were grown in lipid-deficient media in the presence of inhibitors of cholesterol biosynthesis. Control and cholesterol-depleted cells were pulsed with fluorescein isothiocyanate (FITC)-conjugated dextran (F-Dex) or *N*^*α*^-pteroyl-*N*^*ϵ*^-(4′-fluorescein-thiocarbamoyl)-L-lysine (PLF) and A568-labelled Tf (A568-Tf) for 2 min. After washing of excess label, cells were fixed and imaged at either low magnification [0.75 numerical aperture (NA), × 20 objective lens] or high magnification and resolution (1.4 NA, × 60 objective lens) and the fluorescence intensity of endocytosed probe was estimated. After 2 min (data not shown) or 5 min of internalization, F-Dex and PLF uptake was reduced by ∼55%, whereas Tf uptake remained unchanged (within 5–10% of control levels; compare control and depleted cells in Figure 1A; quantification in Figure 1B) in cells depleted of cholesterol.

A closer examination (at higher resolution) of endosomes reveals that cholesterol depletion resulted in fewer number of endosomes formed in 2 min (Figure 1A; quantification in Figure 1E); 43 ± 8 endosomes per cell compared with 20 ± 5 [where the numbers represent weighted mean (wt. mean) and standard error of the mean (SEM), across two experiments]. At this time point, these endosomal structures are likely to represent a mixture of primary endocytic carrier vesicles, the CLICs and the more mature fused endosomal compartments, the GEECs (17). We then quantified the effect of cholesterol depletion on the characteristics of uptake of FR-GPI. Figure 1C (control cells, filled circles; depleted cells, open circles) shows that there is significant reduction in FR-GPI uptake when plotted against the surface receptor expression in cells depleted of cholesterol.

Cholesterol was also depleted using low concentrations of methyl-β-cyclodextrin (MβCD; 2 mM), a water-soluble, cholesterol-sequestering oligosaccharide (23) or filipin (1.5 μg/ml), a polyene antibiotic that complexes cholesterol in cell membranes (24). Both treatments resulted in inhibition of GPI-AP and fluid-phase uptake, whereas TfR endocytosis was unaffected (Figure 1B). To assess the extent of cholesterol depletion after 30-min treatment with MβCD, cells were stained with filipin. This treatment reproducibly reduces filipin staining ∼15–20% (Figure 1D). Repleting cholesterol levels in cells with 2 mM MβCD–cholesterol complexes restores both filipin staining and fluid-phase uptake in cholesterol-depleted cells (Figure 1D), strongly arguing that these effects are because of a specific perturbation of cholesterol and not a secondary effect of MβCD.

**Figure 2:**
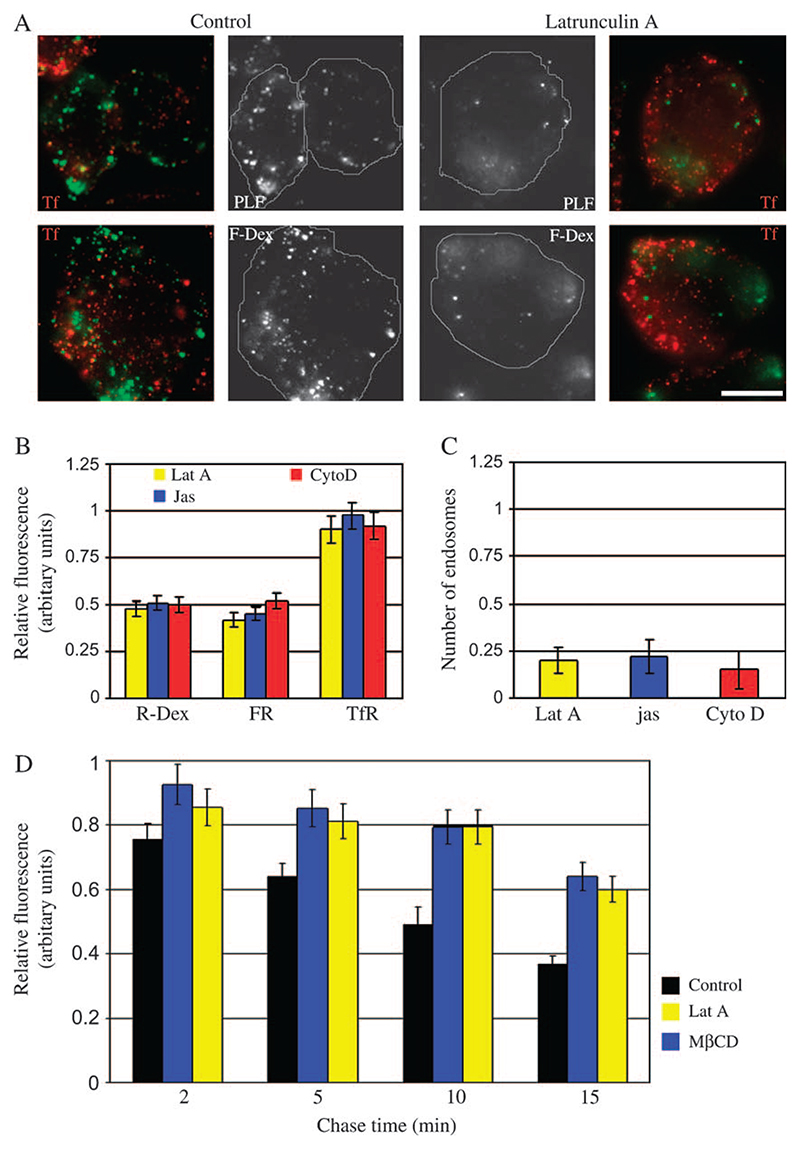
Inhibition of dynamic actin assembly or disassembly blocks endocytic uptake via the GEEC pathway. A) Control (right panel) or Lat A-treated (left panel) FRαTb cells were incubated for 2 min at 37°C with either A568-Tf and PLF (top panel) or A568-Tf and FITC-dextran (lower panel) and imaged at high resolution. Grayscale images of PLF/FITC-dextran (green) and A568-Tf-labelled TfR (red)-containing structures were pseudocoloured and colour merged. Scale bar, 10 μm. B) Histogram shows the extent of uptake after a 5-min pulse of the indicated endocytic tracers in cells subject to Lat A, Jas or Cyto D treatment as described in Materials and Methods, expressed relative to that measured in control cells (value of controls set to 1). Values represented are wt. mean ± SEM obtained across three experiments. C) Histogram shows the average number of GEECs estimated in cells treated with different actin poisons as compared with control cells (value of controls set to 1). Values represented are wt. mean ± SEM across two experiments. D) Histogram shows that regurgitation of fluid is inhibited upon depletion of cholesterol or treatment of cells with Lat A. FRαTb cells were treated with MβCD or Lat A or carrier and pulsed with F-Dex for 2 min and chased for indicated times. Cells were fixed and amount of probe associated with the cells was estimated. Fluid left after the chase was normalized to the amount internalized (initial uptake set to 1). Values represented are wt. mean ± SEM across two experiments.

A possible explanation for decrease in uptake upon cholesterol depletion is faster regurgitation from the newly formed endosomes. To investigate this possibility, cells were treated with MβCD, pulsed with F-Dex and chased for varying times. In control cells, about 40% of endocytosed fluid is regurgitated in 5 min, whereas in cells depleted of cholesterol only about 15% of fluid regurgitates in the same time (Figure 2D). Taken together, the data suggest that cholesterol levels regulate initial uptake of membrane and fluid via GEEC pathway.

### Dynamic actin is essential for the GEEC endocytic pathway

The Rho family GTPases are important regulators of actin polymerization (25) and Cdc42 appears to be specifically required for GEEC endocytosis because dominant-negative (DN) Cdc42 blocks the GEEC pathway of endocytosis whereas DN Rac1 and RhoA have no effect (12). To examine whether the effect of DN Cdc42 on the GEEC pathway was because of reduced actin dynamics, we used pharmacological inhibitors of actin polymerization. Cell-permeant actin polymerization toxins have been used before to inhibit endocytosis via clathrin-coated pits, albeit at higher concentration and longer incubation times and with variable effects (26). Treatment of cells with Lat A, Cyto D or Jas blocks both fluid-phase and FR-GPI uptake, at both 2 min (data not shown) as well as 5 min, to a similar extent as cholesterol depletion, while TfR endocytosis was relatively unaffected (Figure 2A,B); cells treated with actin-binding drugs have lesser number of endosomes as compared with control cells (Figure 2C). Similar to the treatment with MβCD, treatment of cells with Lat A also decreases the extent of regurgitation of fluid phase in cells (Figure 2D), indicating that the effects of actin perturbation on internalization may be more severe than estimated in Figure 2B. These results indicate that the uptake into the GEEC pathway is also strongly modulated by the dynamic status of the actin cytoskeleton.

**Figure 3:**
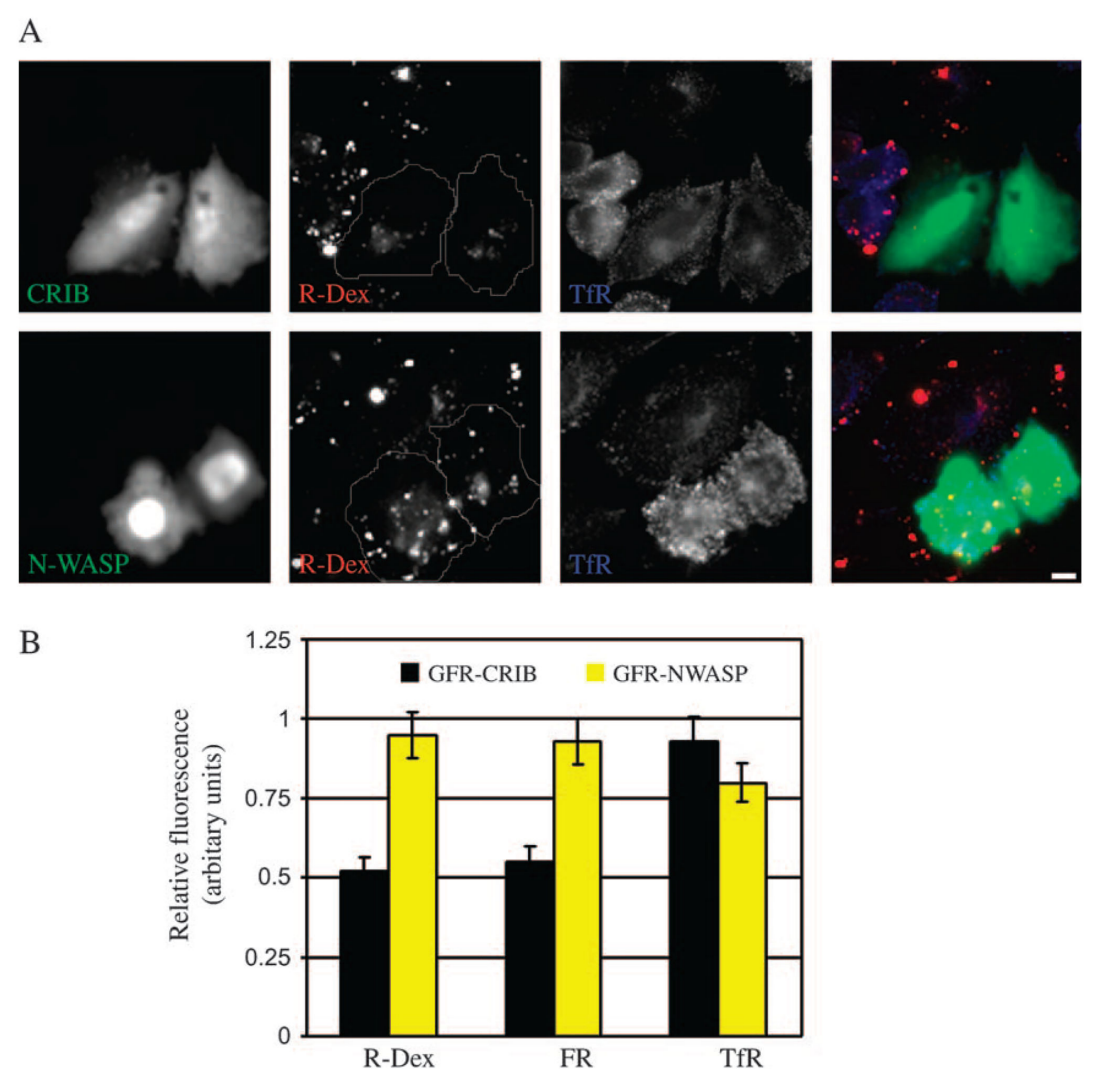
Overexpression of CRIB domain of N-WASP inhibits endocytosis via GEEC pathway. A) FRαTb cells transfected with GFP-CRIB (green; top panel) or full-length GFP-N-WASP (green; bottom panel) were pulsed with R-Dex (red), A647-Tf (blue) for 5 min at 37°C, and imaged at low magnification using a × 20, 0.75 NA objective. Note GFP-CRIB but not GFP-N-WASP-transfected cells show a reduction in fluid-phase uptake relative to non-transfected cells. Scale bar, 5 μm. B) Histogram shows the extent of uptake in a 5-min pulse of indicated endocytic tracers in cells overexpressing GFP-CRIB or GFP-N-WASP, relative to that in non-transfected cells (value of controls set to 1). Values represented are wt. mean ± SEM obtained across two independent experiments.

Coupled with a role for Cdc42 and actin polymerization in GEEC formation, we examined if Cdc42 signalling to the actin cytoskeleton mediated via N-WASP could be involved in this endocytic pathway. To test this hypothesis, we overexpressed the CRIB domain from N-WASP that acts as a DN inhibitor of Cdc42/N-WASP signalling (27). As shown in Figure 3, overexpression of CRIB domain but not full-length N-WASP reduces FR-GPI and fluid uptake (but not TfR uptake), consistent with a role for Cdc42-mediated actin polymerization in the GEEC pathway.

### Cdc42, N-WASP and actin co-localize with nascent GPI-AP and fluid phase-containing endosomes

**Figure 4:**
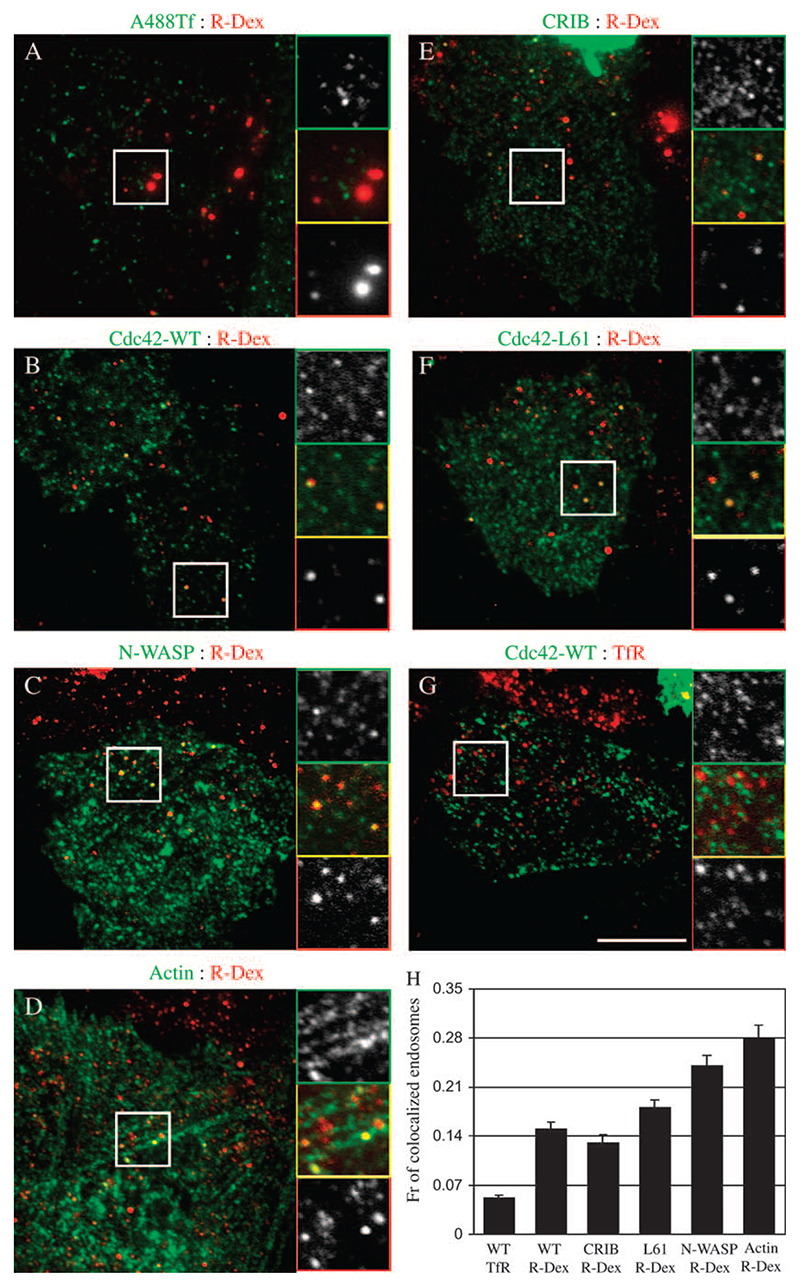
Nascent endocytic carriers co-localize with Cdc42, N-WASP and actin. FRαTb cells expressing GFP-Cdc42 (B and G), GFP-N-WASP (C), actin–GFP (D), GFP-CRIB (E) or GFP-Cdc42-L61 (F) were pulsed with TMR-dextran (R-Dex; A–F) or A568-Tf (G) or A488-Tf (A) for 20 s at 37°C and rapidly washed, fixed and imaged under TIRF illumination (depth ∼100 nm). (H) Histogram shows the extent of co-localization of endosomes labelled with TMR-dextran (R-Dex) or A-Tf (TfR) with expressed GFP-tagged constructs, GFP-Cdc42 (WT), GFP-N-WASP (N-WASP), actin–GFP (actin), GFP-CRIB (CRIB) or GFP-Cdc42-L61 (L61). Extent of non-specific co-localization using the procedures in Materials and Methods is ∼3%. Data were obtained from 10–15 cells, threee to five experiments and represented as wt. mean ± SEM. Scale bar, 10 μm.

To explore a role for Cdc42-regulated actin nanomachinery in the GEEC pathway, we asked whether Cdc42, N-WASP and actin would label endosomes of the GEEC pathway. We started by looking at co-localization of 2-min rhodamine-conjugated dextran (R-Dex)-containing endosomes with green fluorescent protein (GFP)-Cdc42 and found that a very small fraction, less than 3% of 2-min endosomes visualized in wide-field microscope had any GFP-Cdc42 (or N-WASP, actin) labelling on them (data not shown). To find out whether or not Cdc42 associates with early intermediates in the GEEC pathway, such as the pinocytic CLICs, we devised a protocol to preferentially visualize endocytic vesicles that had just budded from plasma membrane and examine their co-localization with cytoplasmically expressed GFP-tagged fusion constructs of Cdc42, N-WASP, CRIB and actin generated and used in previous studies (27,28). In this protocol, cells transfected with GFP-tagged versions of Cdc42, N-WASP and CRIB were pulsed for 20 s with R-Dex and rapidly fixed and imaged using evanescent field of a total internal reflection (TIR) microscope. Control cells show that the majority of R-Dex vesicles are separate from endocytosed TfR (Figure 4A), and completely co-localized with endocytosed GPI-APs (data not shown), confirming their identity as CLICs and/or GEECs. Under these conditions, ∼15% of ‘nascent’ R-Dex-filled endocytic structures co-localize with GFP-Cdc42 (Figure 4B; histogram in H). The extent of co-localization is low but specific for the R-Dex endosomes because <5% of TfR vesicles were decorated with GFP-Cdc42; 18% of R-Dex-positive vesicles were also marked with the dominant-active Cdc42 variant, GFP-Cdc42-L61 (Figure 4F; histogram in H). Under these conditions, however the GFP-Cdc42-N17 mutant was predominantly cytosolic and did not mark any discernable membrane-associated structures, suggesting that activation and deactivation cycle of Cdc42 may be involved in loading of this protein on forming endocytic invaginations. Cdc42/Rac-interactive-binding domain has been shown to bind activated Cdc42 with high affinity (29) and we find ∼13% of R-Dex-containing vesicles labelled with GFP-CRIB (Figure 4E; histogram in H), consistent with the presence of endogenous activated Cdc42 on these endosomes. This finding suggests that GTP exchange cycle of Cdc42 and interaction with its effectors might play an important role in the association (and dissociation) of Cdc42 with these nascent endosomes.

We also explored the prospect of visualizing endogenous Cdc42 staining in CHO cells but met with little success. Although we did see faint antibody labelling on the Golgi complex, we are unable to visualize endogenous Cdc42 staining with R-Dex or TfR-containing endosomes in fixed and permeabilized cells (data not shown). Localizing endogenous Cdc42 has been a challenge and levels of the protein, affinity of available antibodies and staining protocols are important issues to consider (30).

While Cdc42 effectors such as activated Cdc42-associated kinase have been shown to bind clathrin (31), it is not known whether Cdc42 itself associates with clathrin or clathrin-derived vesicles. Less than 5% of endocytosed, Tf-containing vesicles were co-localized with GFP-Cdc42 (Figure 4G; histogram in H), suggesting that Cdc42 may not be retained or recruited to nascent vesicles derived from the clathrin-mediated pathway. We detected a much higher co-localization between N-WASP and actin with the primary endocytic carriers containing R-Dex; >25% of R-Dex-containing vesicles are decorated with these proteins (Figure 4C,D, respectively; histogram in H). Taken together, these results suggest that Cdc42, N-WASP and actin may be recruited to form endocytic buds at the cell surface and retained on the budded vesicle only for a short length of time.

### Ultrastructural identification of membrane domains enriched in GPI-APs and Cdc42

**Figure 5:**
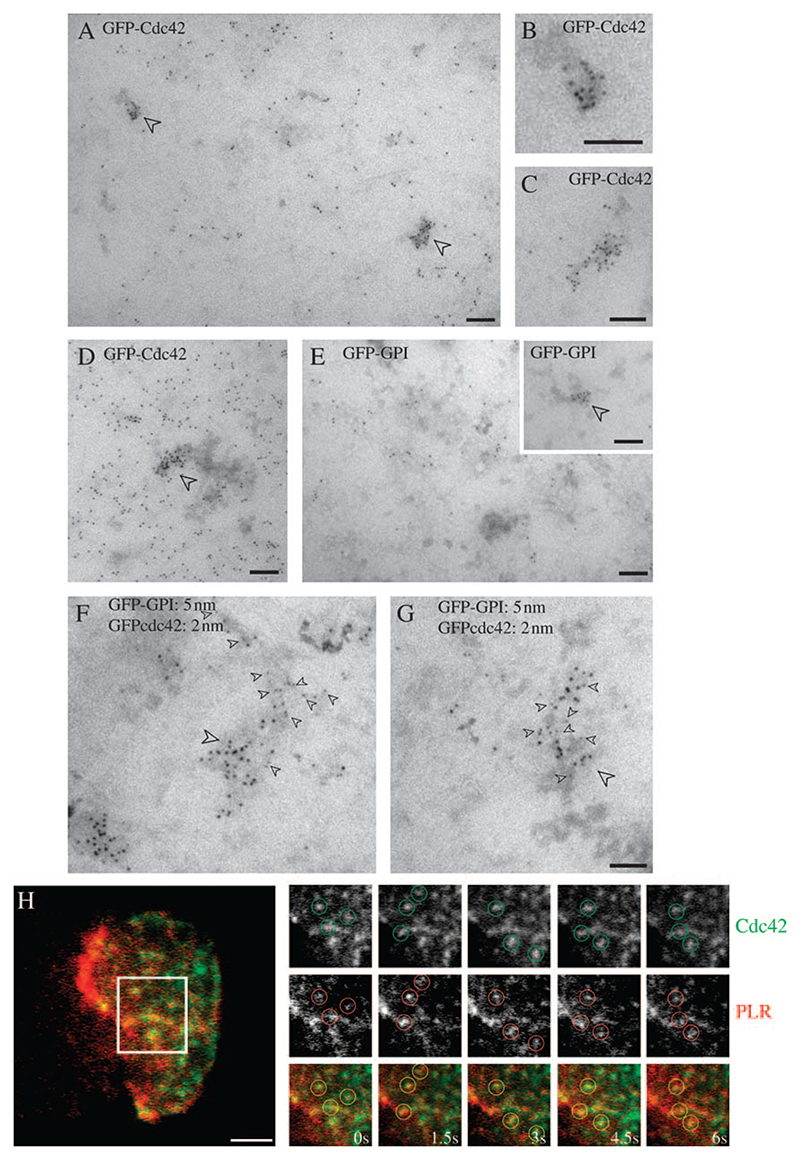
Ultrastructural localization of GFP–GPI and GFP-Cdc42 on plasma membrane. A–G) BHK cells were transfected (A–E) or co-transfected (F–G) with GFP–GPI and/or GFP-Cdc42 as indicated. Cells were pulsed for 2 min with 5 nm anti-GFP-gold antibodies (E–G) prior to making membrane sheets. Membrane sheets were subsequently labelled with 5-nm anti-GFP-gold antibodies (A–E) or 2-nm anti-GFP-gold (F–G). GFP-Cdc42 is distributed over the entire surface but is concentrated in specific areas (arrowheads) as shown at higher magnification in (B) and (C). Note the elongated morphology of the labelled structures (B and C) and how gold particles can be seen to follow a membrane surface in places (e.g. B). In cells expressing higher levels of GFP-Cdc42, similarly sized clusters are still evident (D). Note GFP–GPI label is present over the entire surface (E) with discrete clusters evident in some areas of the cell surface (E, inset). Double labelling of co-transfected cells shows clusters containing both GFP–GPI (large arrowheads) and GFP-Cdc42 (small arrowheads; F and G). Scale bars, 100 nm. H) FRαTb cells were transfected with GFP-Cdc42 and labelled with PLR to mark FR-GPIs on cell surface. Cells were imaged live under dual colour TIRF illumination. Images were acquired at 1.5-s time interval. Montage (taken from Video S1) shows regions in GFP-Cdc42 and PLR image that co-localize with each other. Scale bar, 2 μm.

In order to visualize domains at or near the plasma membrane that may contain GPI-APs and Cdc42 at high resolution, we used electron microscopy on intact two-dimensional plasma membrane sheets. BHK cells were transfected with GFP-Cdc42 alone (Figure 5A–D), GFP–GPI alone (Figure 5E) or were co-transfected with the two constructs (Figure 5F,G). For single-labelling experiments, cells were labelled with 5 nm anti-GFP-gold, either before rip-off, for 2 min at 37°C (labelling surface-exposed GFP–GPI), or after rip-off and glutaraldehyde fixation to label cytoplasmically exposed GFP-Cdc42 as in previous studies (32). In double-labelling experiments, we made use of the fact that GFP–GPI could be labelled before rip-off with 2 nm anti-GFP-gold without labelling of cytoplasmic GFP-Cdc42, and conversely, application of the 5 nm anti-GFP-gold after rip-off only labelled cytoplasmically exposed Cdc42 (as confirmed in double-labelling experiments in which cells were singly transfected for each of the two GFP-tagged constructs; results not shown). Labelling for GFP-Cdc42 was dispersed over the cytoplasmic surface of the plasma membrane but in addition was observed in striking clusters, evident in both high (e.g. Figure 5D) and low (e.g. Figure 5A) GFP-Cdc42-expressing cells. These clusters co-localized with GFP–GPI labelled with anti-GFP-gold on the outside of the cell prior to rip-off (Figure 5F,G), suggesting a co-clustering of these membrane components. These domains were often elongated (mean length 110 ± 15 nm; diameter of 53 ± 12 nm). Interestingly, this is in the size range of the putative primary endocytic carriers, the CLICs (16). Assuming a 42% labelling efficiency for the anti-GFP-gold as calculated previously with the same probes (33), each microdomain contains ∼50 GFP-Cdc42 molecules (52 ± 17).

We also examined whether we could visualize similar structures in living cells. For this experiment, we transfected FRαTb cells with GFP-Cdc42, and labelled FR-GPI with *N*^*α*^-pteroyl-*N*^*ϵ*^-(4′-lissamine rhodamine-thiocarbamoyl)-L-lysine (PLR) at low concentrations, and visualized the two labels in the TIRF field. Consistent with the EM analysis, live-TIRF imaging at 37°C revealed several patches of PLR that were stable over several seconds and enriched for GFP-Cdc42 (Figure 5H; Video S1). It should be emphasized that neither TIRF microscopy nor EM experiments distinguish between cell-surface accessible domains and newly endocytosed vesicles, maintained close to the cell surface. Nevertheless, the structures that co-label for GFP-Cdc42, and PLR-labelled FR-GPI or GFP–GPI (both in the TIRF field and EM, respectively) provide evidence for membranes enriched in both these molecules with respect to the surrounding membrane.

### Dynamics of single molecules of Cdc42 at the cell surface

**Figure 6:**
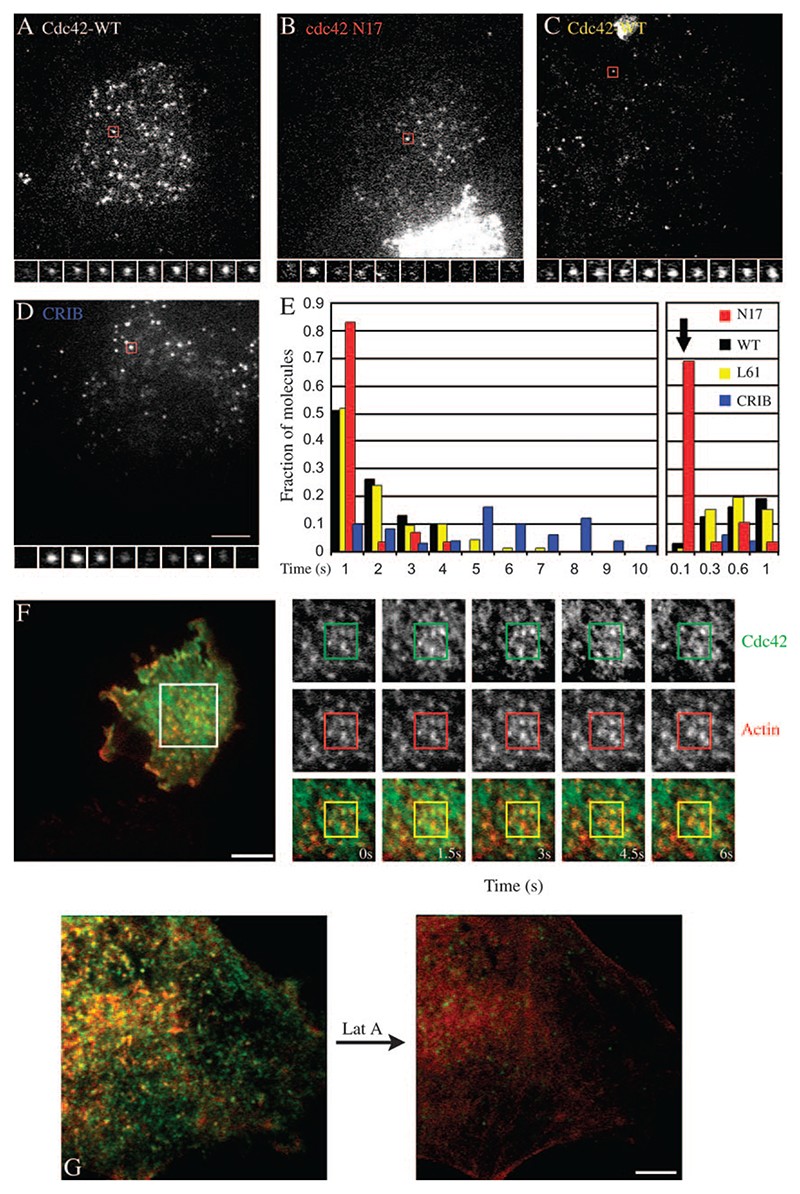
Single-molecule events of Cdc42 and CRIB domain of N-WASP. A–D) FRαTb cells were transfected with GFP-Cdc42-WT, GFP-Cdc42-N17, GFP-Cdc42-L61 or GFP-CRIB DNA constructs, and only cells expressing very low levels of fusion protein were imaged under TIRF illumination with charge coupled device (CCD) cameras. Images and montage taken from videos (Video S4) show single molecules that dwell at a particular subresolution spot indicate long-lived spots in the case of Cdc42-WT and Cdc42-L61 GFP variants, while the GFP-Cdc42-N17 isoform appears only transiently on the membrane. Similarly, stabilized molecules are also demonstrated by GFP-CRIB albeit with long residence times (D). E) Histogram shows the distribution of residence times of Cdc42-WT, Cdc42-L61, Cdc42-N17 and GFP-CRIB DNA molecules on the plasma membrane. Note that a majority of N17 molecules reside for just 100 ms (one frame) in a video and residence time is similar to GFP molecules expressed in cytosol in the same cells (bold arrow); only a small fraction of trajectories exhibit any persistence, and even these show large diffusive trajectories (8–20 cells used for calculation. Each set repeated at least twice). F) FRαTb cells co-expressing Cherry-actin and GFP-Cdc42 were imaged live at 37°C under TIRF illumination, using sequential excitation of both probes. Images and the montage from a video (images were acquired at 1.5-s time; Video 2) show regions where Cdc42 and actin are co-enriched in small dynamic punctate structures. (Scale 10 μm). G) FRαTb cells were transfected with GFP-Cdc42 (green) and Cherry-actin (red) and imaged live, with two-colour TIR illumination (depth ∼100 nm). Cells were treated with Lat A, 4 μM for 2 min, and images of GFP-Cdc42 and PLR was collected before and after treatment. (Scale 5 μm).

To study these plasma membrane regions in detail, we decided to examine the recruitment and dynamics of Cdc42 to the cell surface. For this purpose, GFP-Cdc42 was transfected into cells for a period of 10–12 h and only cells with extremely low levels of GFP fluorescence were observed using a TIRF microscope coupled to back-illuminated Electron multiplying CCD (EMCCD), with the capability to detect single-fluorophore molecules. GFP-Cdc42 appeared as dynamic and punctate fluorescence on the cell membrane (Figure 6A). These punctate structures are single molecules of GFP-Cdc42 on the cell membrane as confirmed by several criteria described in Figure S2.

**Figure 7:**
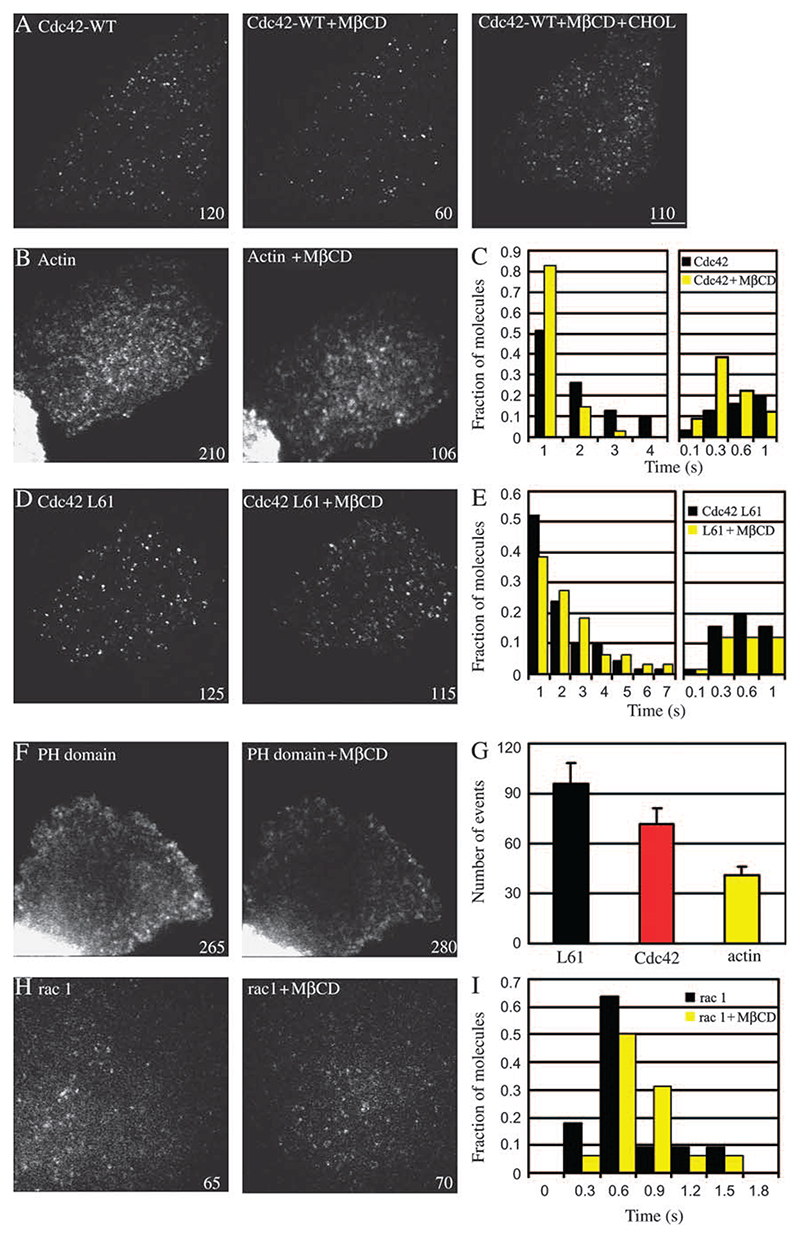
Cholesterol depletion perturbs the recruitment characteristics of Cdc42 and the dynamics of actin polymerization at the cell surface. A–H) FRαTb cells transfected with GFP-Cdc42-WT, GFP-Cdc42-L61, actin–GFP, PH-GFP or GFP-Rac1 were imaged before and after MβCD treatment. A) The number of single-molecule events of GFP-Cdc42-WT decreases upon cholesterol depletion, which can be restored upon addition of cholesterol in depleted cells. C) Histogram shows the residence time of events before (black bars) and after (yellow bars) cholesterol depletion. B) Dynamics of actin decreases upon cholesterol depletion. D and E) L61 mutant of Cdc42 seems refractory to cholesterol depletion. Histogram shows that residence time of events remain unchanged. F, H and I) PH-GFP, Rac1 show little change in event number upon cholesterol depletion. *y*-Axis in all graphs (except G) denoted fraction of molecules and *x*-axis denotes the timescale in seconds. G) Histogram denotes the number of events in treated cases expressed relative to controls (wt. mean ± SEM from two to six experiments). Numbers written at right corner of images denote single molecule events recorded in that cell. Scale bar, 5 μm.

Several controls were performed to examine the dynamics of GFP-tagged molecules in the cell. Cytosolically expressed GFP molecules (pEGFP-N1), rarely visualized in the evanescent field, appear as bright flashes that reside for 100–200 ms in the observation volume. The fluorescence intensity of these events matched with those of GFP molecules settled on a coverslip (Figure S2A,E). In contrast, GFP molecules tagged with pleckstrin homology (PH) domain of phospholipase C delta, tagged with GFP, PH-GFP (34), are recruited to the membrane quite efficiently but mostly show diffusive motion (Figure 7F). GFP-Cdc42 is also recruited to the plasma membrane quite efficiently and besides displaying diffusive motion it localizes in subresolution spots in the plasma membrane for 3–5 s (Figure 6A,E). The relatively static nature of GFP-Cdc42 in the membrane indicates that it resides on a relatively stable structure in the cell membrane, presumably mapping to those visualized in the EM and TIRF field (Figure 5). The sites to which GFP-Cdc42 is stably recruited to the plasma membrane appear enriched in stable actin structures as visualized by dual colour TIRF microscopy (Figure 6F; Video S2).

Upon treatment of cells with Lat A, the elaborate cortical actin network becomes featureless although the general cell morphology is still intact (Figure 6G). After Lat A treatment, the intensity and distribution of GFP-Cdc42 undergo significant changes; the number of Cdc42-rich regions at or close to the plasma membrane decreases drastically. These results suggest that continued actin polymerization is required for recruitment of Cdc42 at these actin-rich sites.

The behaviour of the wild-type GFP-Cdc42 (WT Cdc42) seems to be a function of the activation status of the molecule, Cdc42, because the GTPase-deficient mutant, GFP-Cdc42-L61, displays a similar pattern of activity and is present on the membrane for a much longer duration (Figure 6C), while GTP-binding-deficient GFP-Cdc42-N17 is rarely recruited to single stable foci in the membrane (Figure 6B) and resembles cytosolically expressed GFP (Figure S2G). We then used the CRIB domain tagged with GFP to detect the activated form of endogenous Cdc42 (28). We expressed GFP-CRIB at low levels of expression and examined its dynamics. Similar to Cdc42, the CRIB domain is recruited to the plasma membrane and single molecules of GFP-CRIB are stabilized for 4–10 s, much longer residence times than either wild-type or the GTPase mutant (Figure 6D; Video S4). This is consistent with the observed stabilization of the GTP-bound state of Cdc42 by binding of the CRIB domain *in vitro* (29). These results support the hypothesis that Cdc42, once activated, is stably localized to actin-enriched domains in the cell surface (Video S2).

### Cdc42 recruitment and activation, and actin dynamics are sensitive to cholesterol depletion

To understand the effect of mild cholesterol depletion on the operation of the GEEC pathway, the activity of single molecules of GFP-Cdc42 was measured before and immediately after cholesterol depletion. It should be noted that at these moderate levels of cholesterol depletion, it was difficult to detect any gross perturbation in the levels of Cdc42 recruited to the cell membrane, given the variability of expression levels of the GFP-Cdc42 in a population of transfected cells (data not shown). Because of ease and duration of protocol, we used MβCD treatment to deplete cells of cholesterol. Mild cholesterol depletion using MβCD (2 mM) results in a consistent decrease in fluid and FR uptake and is completely reversible (Figure 1B,D). The total number of Cdc42 molecules that were stably localized at the plasma membrane (events) as well as their residence time was quantified as described in Materials and Methods.

Cholesterol depletion had a significant effect on the number of Cdc42 molecules recruited to the cell membrane; ∼35–40% reduction in cells depleted of MβCD (Figure 7A,G). There is also a discernable change in the residence time of the remaining events (Figure 7C). Re-addition of cholesterol to the same cell restored the recruitment of Cdc42 (Figure 7A; Video S5); 70% of cells showed almost complete (90%) recovery in terms of their ability to recruit Cdc42 molecules to the plasma membrane, consistent with a requirement for adequate cholesterol levels in the cell membrane for these processes (see also Figure 1D).

We next examined the dynamics of the GTPase-deficient GFP-Cdc42-L61 isoform upon mild cholesterol depletion. To our surprise, both the number and residence time of GFP-Cdc42-L61 were much more resistant to cholesterol depletion than WT Cdc42 (Figure 7D,E and G; Video S6). This suggested to us that endogenous levels of cholesterol are important for efficient GTP exchange on the plasma membrane pool of Cdc42. To confirm that the inhibition of GFP-Cdc42 recruitment upon mild cholesterol depletion is indeed mirrored by inhibition of endogenous Cdc42 activation and recruitment, we examined the effect of mild cholesterol depletion on the dynamics of the activated Cdc42-binding CRIB domain. This treatment significantly decreased the recruitment of GFP-CRIB domain in a fraction of cells examined (∼40% fewer events were detected after cholesterol depletion; data not shown) consistent with a role of cholesterol depletion in altering the dynamics of endogenous Cdc42 at the plasma membrane.

As a control for the specificity of the effects of cholesterol depletion, we examined recruitment and dynamics of GFP-Rac-1 (Video S8), PH-GFP (Video S9) (Figure 7F,H and I), and dynamin 2aa-GFP levels (Figure S3C,D; Video S10) on the plasma membrane, before and after similar MβCD treatment. Single molecules of GFP-Rac1 were stably recruited to the plasma membrane for about a second and neither the number of molecules nor their residence times were sensitive to mild cholesterol depletion. Similarly, mild depletion of cholesterol had no effect on the number of events of PH-GFP, suggesting that the turn over of phosphatidylinositol (4,5)-bisphosphate (PI(4,5)P2) is unaffected. However, complexing large amounts of cholesterol from cells, through treatment with filipin, led to a reversible redistribution of PH-GFP to the nucleus (see Figure S3A) consistent with previous observations by Edidin and co-workers (35). Dynamin 2aa-GFP exhibited distinct, fluorescent spots on plasma membrane, which were resident for about 20 s (mean residence time), coinciding with time of generation of clathrin-coated vesicles (36). There was, however, no discernable effect of mild cholesterol depletion on the dynamics and recruitment of dynamin 2aa-GFP on plasma membrane, consistent with the lack of an effect of this treatment on endocytosis via the dynamin-dependent, clathrin-mediated pathway.

Inhibition of Cdc42 activation by cholesterol depletion could result in an effect on cortical actin dynamics in cells. To address this question, we first ascertained the distribution of phalloidin-stained F-actin in cells treated with increasing MβCD concentrations. At high concentrations (10 mM MβCD), we noticed a dramatic redistribution of actin filaments near the plasma membrane; stress fibres were also quite perturbed (Figure S3B). These results are also similar to those reported by Edidin and co-workers (35). However, under conditions where endocytosis via GEECs is specifically affected (at low MβCD concentrations), the phalloidin-stained actin cytoskeleton is not detectably altered (data not shown). To examine more closely the effects of low levels of cholesterol extraction, we investigated the dynamics of actin at the plasma membrane with TIRF illumination. At low levels of expression, actin–GFP appears as punctate structures in untreated cells (Figure 7B). The majority of these puncta are dynamic in nature with residence times ranging from 1 to 7 s (data not shown). Upon depletion of cholesterol, there is not only a substantial reduction in number of events that were recorded but also the majority of actin puncta became stationary (see Video S7). This is consistent with the effects of cholesterol depletion on Cdc42, with Cdc42 playing a role of catalyst to initiate actin polymerization in the cell cortex.

## Discussion

### Cholesterol levels, GPI-AP nanoclusters and endocytosis into GEECs

We have recently shown that GEECs are formed by the fusion of primary dynamin, clathrin and caveolin-independent endocytic carriers called CLICs (16,17), and maintain their identity for a short period (∼2–5 min), before acquiring Rab5 and EEA-1 and fusing with sorting endosomes derived from the clathrin-coated pit pathway (17).

To understand the initial steps in the formation of the earliest endocytic vesicles that give rise to the GEEC endosomal system, we have explored the role of cholesterol. We have shown that, while acute cholesterol depletion (10 mM MβCD) disrupts nanoclusters of GPI-APs, treatment of cells with 1–2 mM MβCD (mild conditions), did not detectably alter their presence in cell membranes (19). Mild cholesterol depletion, however, resulted in a dramatic reduction in the number of detectable endosomes per cell. Also, the decrease in uptake of fluid and endosomal compartments cannot be accounted for by enhanced regurgitation; cholesterol depletion leads to slower recycling of internalized fluid. Constitutive recycling of fluid from cells has been studied before, but there are little insights into its mechanisms (37). These results strongly suggest that mild cholesterol depletion may specifically reduce the frequency of primary endocytic events involved in internalization via this pathway.

Our results suggest that different cholesterol thresholds in cell membranes play distinct roles in endocytosis via the GEEC pathway, one in organizing GPI-AP clusters (19) and the other in influencing their endocytosis.

### Cdc42-dependent actin polymerization and GPI-AP recruitment

Cdc42-dependent actin polymerization appears to be a key component of the GEEC pathway. As shown previously, this pathway is inhibited by expression of the GTP-binding-defective Cdc42-N17 mutant as well as by treatment with the *Clostridium difficile* toxin B, a specific inhibitor of Rho GTPases (12,15). To further the understanding of GEEC pathway, we now show that a range of fungal metabolites that have a perturbing effect on actin dynamics. Lat A, Cyto D and Jas, selectively inhibit both fluid phase and GPI-AP uptake and reduce the number of endosomes; actin perturbation also inhibits regurgitation of cargo endocytosed via this pathway at early times. The estimated ∼50% decrease in fluid uptake (Figures 1B and 2B) upon cholesterol depletion and treatment of cells with actin poisons is an underestimate keeping in mind that these treatments also decrease the rate of regurgitation of fluid. Therefore, similar to the role of cholesterol, actin polymerization also seems to regulate the frequency of primary endocytic events involved in internalization via this pathway. Consistent with the role of Cdc42-based signalling in the actin polymerization system, N-WASP appears to be involved in the GEEC pathway because expression of CRIB domain [CRIB motif of N-WASP; (27)] acts as a DN suppressor for uptake via GEECs.

Total internal reflection fluorescence microscopy is ideally suited to visualize endocytic events occurring in an ∼100-nm swathe of cell cytoplasm close to the glass coverslip (38). Using a TIRF microscope coupled to sensitive cameras, we have been able to visualize endosomes that have recently formed. These structures have all the characteristics of CLICs; they are separate from endocytosed Tf, and contain a significant amount of the fluid phase and co-localize with GPI-APs (data not shown). Importantly, constitutively activated Cdc42, N-WASP and actin decorate these nascent endosomes when GFP-tagged isoforms are expressed at very low levels inside the cytoplasm of cells. Consistent with the above finding, CRIB domain also labels these newly formed endosomes.

Further confirmation of this link comes from the visualization of the dynamics of Cdc42 and actin patches at or near the cell surface. In live cells in the evanescent field, actin is enriched at specific sites that have a high Cdc42 density. While we have not done simultaneous imaging of all three proteins, GPI-APs, Cdc42 and actin are likely to coexist in these patches (Videos S1 and S2). These results support the proposal that Cdc42-dependent actin recruitment to the cell membrane may be involved in initial steps in endocytic vesicle formation.

At the ultrastructural level, Cdc42 showed extensive co-localization with clustered GPI-anchored GFP, and TIRF microscopy confirmed that concentrated GPI-APs at or near the cell surface are indeed associated with stabilized Cdc42 structures. Currently, the mechanism of formation of segregated GPI-AP domains is under investigation (manuscript in preparation). Given that the morphological characteristics of the Cdc42 clusters were very similar to those of putative early carriers (CLICs) in the GEEC pathway, which were identified in electron microscopic studies (16), these results locate Cdc42 squarely in the membrane invagination process in this endocytic pathway.

Recent data from Drubin’s laboratory elegantly demonstrated that endocytosis in *Saccharomyces cervesiae* starts at cortical actin patches on the plasma membrane. These actin-rich patches contain proteins involved in receptor-mediated endocytosis and appear to be defined and initiated by clathrin, but soon, proteins that regulate (Las17p and Arp2/3) actin take over the process and participate actively in vesicle formation and pinch off event (39). In fact, clathrin is dispensible in this process. It is tempting to speculate that similar mechanisms play a role in the Cdc42-regulated endocytosis described here; actin patches and stabilized Cdc42 molecules visualized in live imaging are remarkably similar to actin patches seen at the yeast cell surface.

Rho GTPases have been broadly implicated in both clathrin-dependent and independent endocytosis (1,40) and phagocytosis and processing of bacterial pathogens (41). Cdc42, specifically, has been implicated in regulating fluid uptake in dendritic cells in a developmentally regulated manner (42). Cdc42 also plays a primary role in compensatory endocytosis in fertilized oocytes; rapid membrane re-uptake to compensate for the secretion via cortical granules (28). Thus, understanding the dynamics of Cdc42 activation at the cell membrane will have implications for a number of membrane invagination processes. At this time, further experimentation at the ultrastructural level, supported by TIRF microscopy, are necessary to explore the nature of this connection. The ability of Cdc42 to remodel the actin cytoskeleton is expected to play an important part in this mechanism.

### Cholesterol sensitivity of Cdc42 activity at the plasma membrane

Cholesterol depletion from cell membranes using MβCD has been previously shown to affect cell shape and actin cytoskeleton via redistribution of (PI(4,5)P2) in cells (35). Changes in the actin cytoskeleton that have been observed previously are consistent with changes in the activity of actin-modifying proteins that are regulated by PI(4,5)P2 (35,43). Consistent with these results, we also find that acute and large-scale cholesterol depletion disrupts the organization of the actin cytoskeleton and alters levels of PI(4,5)P2 in cells as visualized by a loss of PH domain from the plasma membrane (Figure S3). In contrast, under the conditions of mild cholesterol depletion, PH domain recruitment to the cell surface seems unaffected, and global actin cytoskeleton organization seems rather unperturbed. However, the dynamics of the actin cytoskeleton near the plasma membrane (as visualized in the TIRF field) is significantly altered (Figure 7; Video S7).

Understanding the dynamics of different GTP-bound states of Cdc42 with respect to its behaviour at the plasma membrane could provide an explanation for the effects of cholesterol depletion. Our studies in control cells show that WT Cdc42 is recruited and stabilized at the membrane in structures that contain dynamic actin. Furthermore, the dynamics of the activated (GTPase deficient) mutant and the dynamics of the GFP-tagged CRIB domain strongly suggest that plasma membrane recruitment is accompanied by Cdc42 activation and stabilization. Mild cholesterol depletion specifically affects dynamics of WT Cdc42 on plasma membrane in terms of the number of sites of Cdc42 attachment and residence time for the recruited molecule, while the dynamics of GTPase-deficient Cdc42-L61 proved to be refractory to cholesterol depletion. In contrast, GTP-binding-deficient Cdc42-N17 is rather poorly recruited to the plasma membrane. Therefore, we conclude that cholesterol depletion inhibits both the activation of Cdc42 and its consequent recruitment/stabilization in the plasma membrane. This effect is more likely to be because of cholesterol-sensitive activity of a guanine nucleotide exchange factor (GEF), potentially by altering the local composition of lipids in plasma membranes. This is a rather specific effect because the dynamics of a different Rho family member, Rac1, is unaffected by this perturbation. The dynamics of another GTPase, dynamin, involved in clathrin-mediated endocytosis, is also unaffected by this mild level of cholesterol depletion, consistent with the lack of an effect on uptake of Tf by the clathrin-mediated endocytic pathway at these levels of cholesterol depletion.

Rho GTPases act as molecular switches that cycle between active (GTP bound) and inactive (GDP bound) conformations. GTPase-activating proteins (GAPs) enhance the intrinsic slow rate of GTP hydrolysis while GEFs catalyze the exchange of GTP for GDP. Activation of these key regulators in localized regions in cell often determines spatial-temporally regulated function of GTPases (44). *dbl* was the first gene to be discovered with nucleotide exchange activity for Cdc42 (45). While many GEFs are promiscuous, some like Fgd 1 show strong preference towards Cdc42 (46). Fgd1 is localized in the actin-rich cell cortex in several mammalian cell lines and regulates activation of Cdc42. In the context of the operation of the GEEC pathway, it would be important to determine how these GEFs and GAPs are modulated by membrane cholesterol.

Recently, Pagano and co-workers (47) have shown that sphingolipid depletion perturbs the membrane targeting of Cdc42 and RhoA. These studies also showed that altering sphingolipid levels in the cell membrane perturbs uptake via the Cdc42-sensitive fluid pathway. Along with the observations detailed here, addressing the role of cholesterol in the recruitment of Cdc42 to the cell surface, we propose that Cdc42 recruitment and activation could occur in specialized sphingolipid- and cholesterol-rich membrane regions, alternatively is accompanied by the construction of specialized domains that require both sphingolipid and cholesterol. These domains could then concentrate GPI-APs at the outer leaflet, potentially by recruiting cholesterol-sensitive nanoclusters, as suggested earlier (21).

In conclusion, our data suggest a mechanism that couples cholesterol levels directly with the regulation Cdc42 activity at the cell membrane. These results provide strong evidence that endocytosis via the GEEC endocytic pathway is directly governed by a cholesterol-sensitive Cdc42-based actin nanomachinery at the cell surface.

## Materials and Methods

### Materials

PLF and PLR are fluorescent folic acid analogs and were used to mark FR-GPI in cells. Fluorescein isothiocyanate-conjugated dextran or TMR-dextran (R-Dex) from Invitrogen Molecular Probes (Carlsbad, CA, USA), were used as fluid-phase tracers. Transferrin was purified as described before (48) and conjugated to Alexa-568 (A568-Tf) or Alexa-647 (A647-Tf) reactive dyes and used at saturating concentration to mark TfR. All other chemicals and media reagents were obtained from Sigma-Aldrich (St Louis, MO, USA) or Gibco Invitrogen (Carlsbad, CA, USA) unless otherwise specified. All fluorescent reagents and probes were dyes from Invitrogen Molecular Probes or GE Health sciences–Amersham Biosciences (Cy5 labelling kit; Piscataway, NJ, USA) and have been described earlier (12,22,49). OKT-9 is a monoclonal antibody against human TfR. OKT-9 hybridoma was grown; antibody harvested using a protein-G column and conjugated to Alexa-647 probe. Folate-free Ham’s F12 (HF-12) was obtained from Hi-Media (Mumbai, Maharashtra, India), Fugene 6 and Nutridoma-SP (serum-free media supplement) from Roche Applied Science (Indianapolis, IN, USA). Latrunculin A and Cyto D were purchased from Sigma-Aldrich and Jas from Invitrogen Molecular Probes. Filipin III and cholesterol–MβCD complex (Cholesterol–water soluble) was purchased from Sigma-Aldrich. Dyn2aa-GFP was kindly provided by Mark McNiven (50). PH-GFP was a gift from Tobias Meyer. GFP-Cdc42 and its mutant isoforms, GFP-N-WASP, and GFP-CRIB domain were kindly provided by Mike Way. Actin–GFP was purchased from Clonetech Inc. (Mountain View, CA, USA).

### Cell culture and treatments

Glycosyl-phosphatidylinositol-anchored folate receptor-expressing CHO cells (FRαTb) were maintained as described previously (22,49). For metabolic depletion of cholesterol, FRαTb cells were seeded at different densities in coverslip bottom dishes (to match cell density at the time of experimentation). Twenty-four hours after seeding, adhered cells were washed with PBS and allowed to grow in either control medium (HF-12 with 10% Heat inactivated FBS) or cholesterol depletion medium (HF-12 with 1% dialyzed nutridoma; compactin, 10 μM; mevalonic acid salt, 200 μM and BSA–oleic acid, 10 μM) for 48 h. Nutridoma-SP was dialyzed to remove folic acid.

Cholesterol was also depleted under mild conditions from cells by MβCD (2 mM for 30 min at 37°C) or filipin (1–1.5 μg/ml for 15 min at 37°C). Cholesterol levels in cells were restored by incubating them with MβCD–cholesterol complexes (2 mM) for 20 min at 37°C.

For perturbing polymerization of actin, cells were treated with Lat A 5 μM, Cyto D 6 μM or Jas 4 μM or carrier (dimethyl sulphoxide) for 5 min at 37°C prior to analysis of endocytic capacity. Note that the dose of actin perturbants as well as extent of lipid depletions were carefully titrated to specifically study perturbations on GPI-AP endocytosis (Figure S1). Under the conditions used in this manuscript, unless specifically mentioned, TfR endocytosis was not detectably affected.

### Labelling of probes and endocytic assays

To label endosomes, cells were incubated with specific endocytic tracers, for the indicated times in labelling medium (HF-12, 10% serum) or medium 1 [M1 (48); with 0.1% glucose]. In some experiments, to give endocytic pulses shorter than a minute, cells were labelled with PLF or A568-Tf on ice for 1 h and instantly warmed up to 37°C by placing coverslip dishes on a pre-warmed water bath.

For fluid regurgitation experiments, FRαTb cells were treated with MβCD (2 mM) or Lat A (5 μM) or carrier and pulsed with F-Dex for 2 min. After washing excess probe, cells were kept back at 37°C and the initial F-Dex pulse was chased for 2, 5, 10 or 15 min. At the end of chase period, cells were fixed and imaged and amount of probe associated with the cells was estimated. Uptake in treated cells as shown in Figure 1 is sensitive to the treatments employed. Histogram in Figure 2D shows amount of fluid remaining in cells after respective chase periods, in treated or control cells, expressed relative to the initial uptake.

Fluorescently labelled Fab fragments of Mov18 and Tf were prepared as described previously (22,49). Fluorescein isothiocyanate-conjugated dextran or TMR-dextran (R-Dex) was used at 1 mg/mL to mark the fluid-phase uptake. After incubation with labelled tracers, excess label was washed off with multiple washes of chilled M1. To remove surface receptor-bound ligands (A568-Tf and PLF), cells were treated with chilled ascorbic acid buffer (pH 4.5, 10 min) or salt-balanced glycine–HCl buffer (pH 2.5, 30 s). Cells were fixed with 2% paraformaldehyde or ATP depletion cocktail carbonyl cyanide m-chlorophenylhydrazone (CCCP, sodium azide, 2-deoxy glucose) in M1.

Cells were transiently transfected using Fugene 6 according to manufacturer’s instructions. To estimate the decrease in cholesterol levels, cells were stained with filipin as described in Mukherjee et al. (51). Briefly, cells were fixed and blocked using Glycine-M1 50 mM and stained with filipin solution (50 μg/ml) for 2 h. Cells stained with filipin were imaged using × 380–440m filter cube from Chroma Technology Corporation (Rockingham, Vermont, USA).

### Quantitative wide-field fluorescence imaging and quantification procedures

Wide-field fluorescence imaging was carried out using a Nikon TE 300 inverted microscope equipped with × 60, 1.4 NA or × 20, 0.75 NA objective (for high resolution or quantitative imaging respectively), using a mercury arc illuminator (Kawasaki, Kanagawa, Japan). Images were collected using a cooled CCD camera (TEK-512B CCD detector mounted in a MicroMax Camera (Princeton Instruments, Monmouth Junction, NJ, USA), controlled by METAMORPH software (Molecular Devices, Sunnyvale, CA, USA). Optimized dichroics, excitation and emission filters were used as described (49).

As there is considerable cell to cell variability in the expression of FR-GPI and TfR (∼ 40% variability in TfR expression), to measure the amount of endocytosed FR-GPI or TfR, in control or treated cells, internalized PLF or A568-Tf fluorescence from individual cells was normalized against surface-bound PLR or Cy5-OKT-9, respectively. In each experiment, normalized fluorescence is estimated from ∼150 cells from duplicate dishes (about 300 cells in total per data point). Weighted mean and SEM were estimated from three to five such experiments and expressed relative to control samples (when controls are set to 1).

### Quantitative co-localization analysis

The quantification of co-localization of endocytic markers in high-resolution wide-field images was determined using established software routines capable of identifying and quantifying the intensity of each endosome, followed by determining the extent of co-localization of individual endosomes with protein markers in a reference image (48,52,53). Co-localization index was calculated as a ratio of the co-localized intensity to the total intensity of the probe in endosomes identified in each cell. Co-localization index is represented as a wt. mean and SEM obtained from two to three independent experiments from at least 10–15 cells in each experiment (12).

### Single-molecule TIRF imaging

TIR microscopy (TIRF) imaging was done on a custom-built TIRF setup around a Nikon Eclipse TE 2000U inverted microscope setup (Kawasaki, Kanagawa, Japan, and supplied via Towa Optics, New Delhi, India). Argonion (488 nm) and helium–neon laser (543 nm) were coupled and launched into optic fibre feeding into the microscope. Using focusing lenses, the lasers were focused onto back focal plane of a high NA × 100 objective (1.45) to get parallel beam hitting the sample plane. The angle of incidence of lasers is changed using micrometre screws, and at angle higher than critical angle (around 65°), TIR occurs. The depth of penetration of evanescent wave was determined to be around 90–100 nm for 488 nm excitation as determined by comparing the diameter of a 1.2-μm fluorescent bead when imaged by wide-field or TIRF illumination (Figure S2).

Images were collected using appropriate filters onto cooled EMCCD-based Cascade 512B cameras (Roper Scientific, Tucson, AZ, USA) with ’on-chip multiplication gain’ feature for providing single-molecule detection sensitivity in solutions and cells. For two-colour TIRF microscopy, cells were illuminated by 488 or 543 nm lasers and fluorescence emission was collected using corresponding emission filters mounted on a shutter wheel (Sutter instrument, Novato, CA, USA) operating coordinately with the illuminating lasers.

Cells expressing low levels of GFP-Cdc42 (or other GFP fusion proteins) were imaged under illumination from evanescent field of 488-nm argon-ion laser with appropriate filters sets. Cells were maintained at 37°C in live-cell imaging media (0.1% glucose and 0.2% serum) using a thermocouple-based heated stage. Videos of single molecules of GFP-Cdc42 were recorded at frame rate of 12 fps (10 ms exposure) for about 10–12 s.

Recruitment of single molecules at plasma membrane was marked as an event and the residence time of each event was followed through frames either manually or using ’motion track’ routine in METAMORPH. Timescale of several events (typically 100–200 from a cell) are combined and plotted as a distribution of residence time versus fraction of events. Typically, data from two dishes (4–5 cells/dish) are combined to give rise to frequency distributions. Frequency distributions of residence time of single molecules and their number are estimated before and after cholesterol depletion from each cell, and data averaged after normalizing to the number of events prior to cholesterol depletion.

### Electron microscopy

BHK cells were co-transfected with GFP–GPI and GFP-Cdc42. Five nanometers gold-conjugated anti-GFP antibodies were bound to live cells at 4°C for 10 min to label GFP–GPI. Cells were warmed to 37°C for 2 min to allow internalization into GEEC. Plasma membrane sheets were prepared as previously described (32) and stained with 2 nm anti-GFP-gold antibodies (for double-labelling experiments) or 5 nm anti-GFP-gold (for single labelling) to label GFP-Cdc42. GFP–GPI or GFP-Cdc42 individually transfected cells were labelled with both probes, exactly as above, to check for cross-labelling of gold-conjugated anti-GFP antibodies. Electron microscopy and other processing steps were as described previously (32,33).

## Supplementary Material

movie 1

movie 2

movie 3

movie 4

movie 5

movie 7

movie 8

movie 9

movie 10

Supp Fig1

Supp Fig2

Supp Fig3

Supplementary Figures and Movies Legends
