# Supplementary figures and images for: Cholesterol-Sensitive Cdc42 Activation Regulates Actin Polymerization for Endocytosis via the GEEC Pathway
Source: Traffic. Author manuscript; Available in PMC 2024 Dec 9. (PMC7617178; doi:10.1111/j.1600-0854.2007.00565.x)

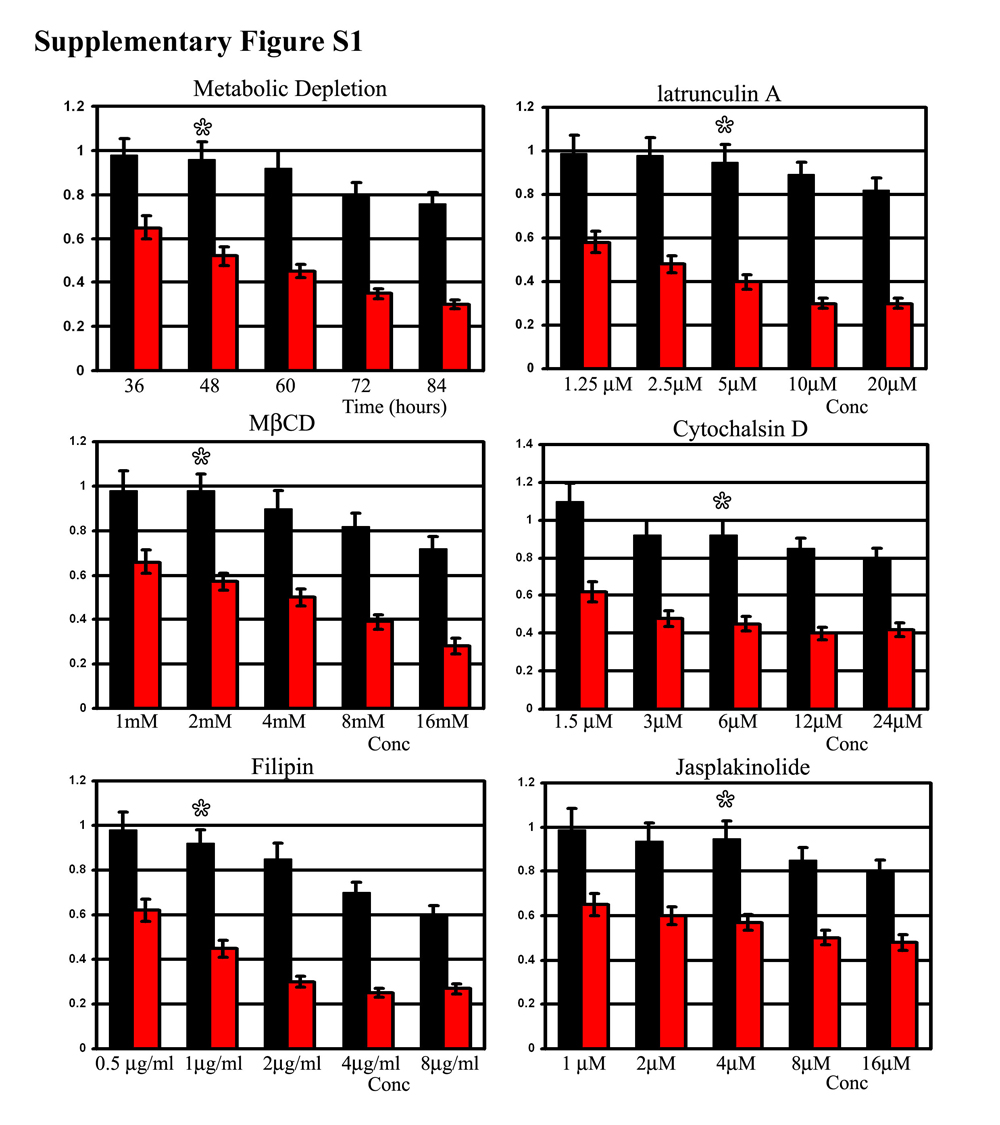

Supplement: Supp Fig1 [file EMS198606-supplement-Supp_Fig1.jpg]

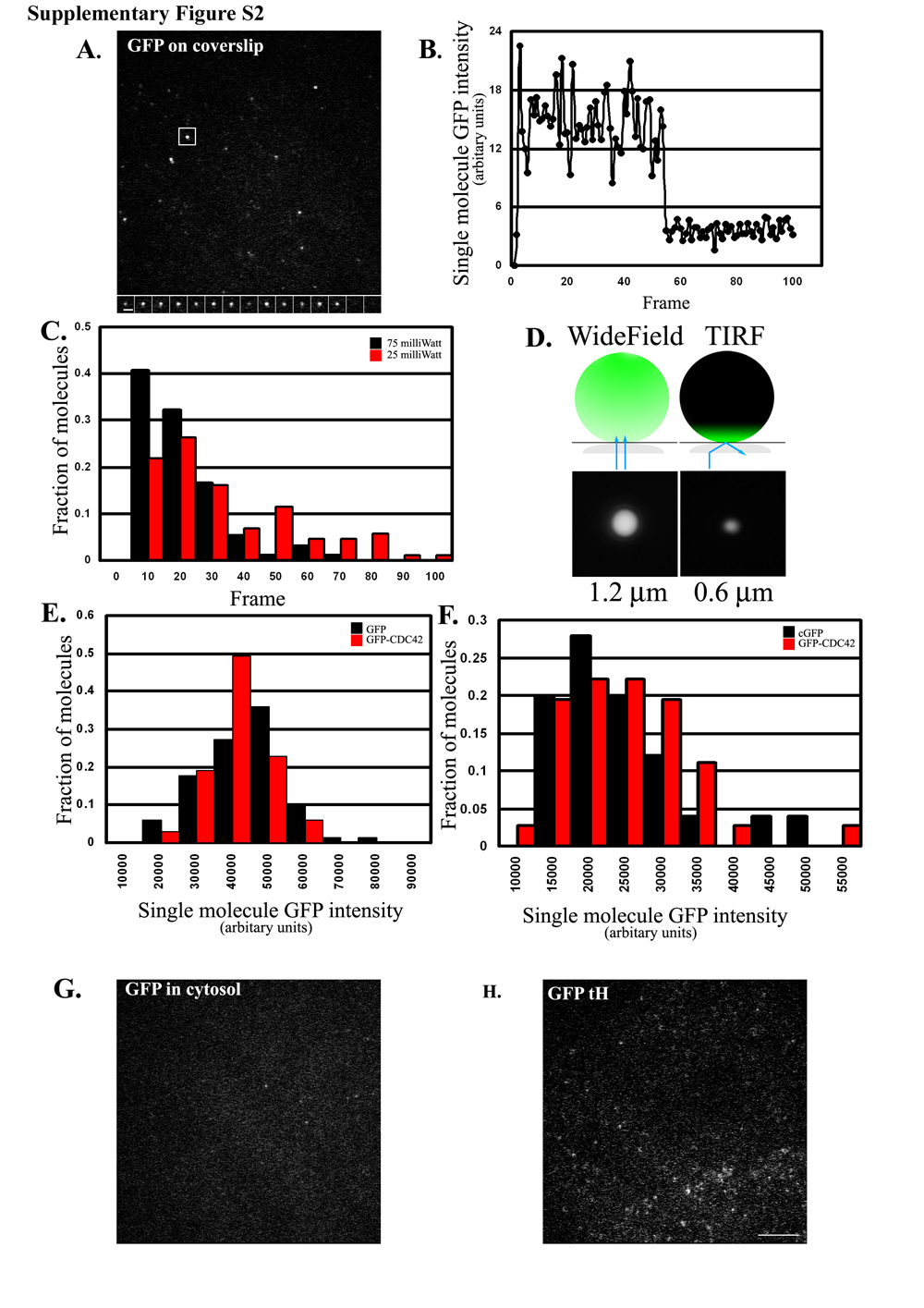

Supplement: Supp Fig2 [file EMS198606-supplement-Supp_Fig2.jpg]

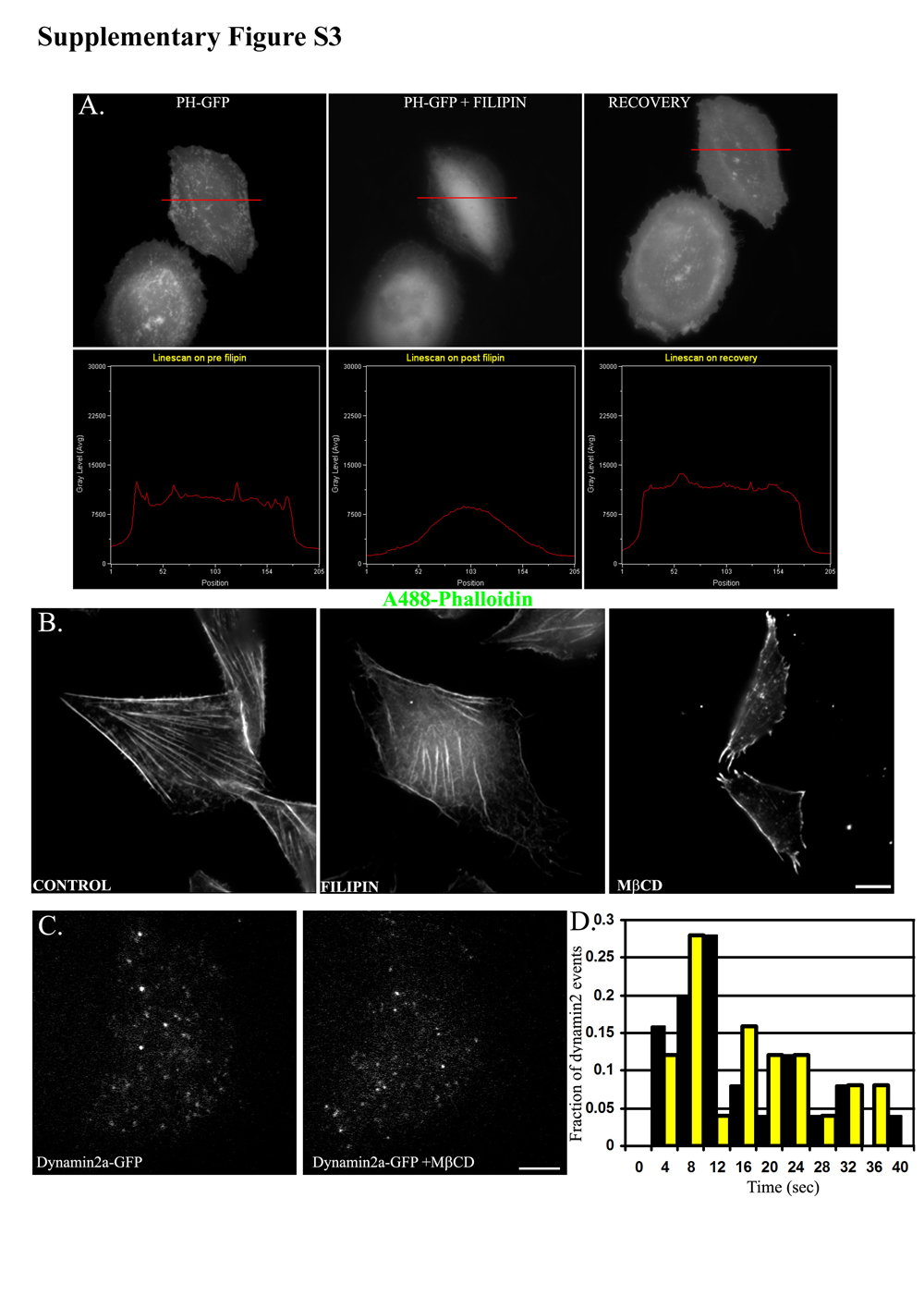

Supplement: Supp Fig3 [file EMS198606-supplement-Supp_Fig3.jpg]
