# Supplementary material for: Cholesterol-Sensitive Cdc42 Activation Regulates Actin Polymerization for Endocytosis via the GEEC Pathway
Source: Traffic. Author manuscript; Available in PMC 2024 Dec 9. (PMC7617178; doi:10.1111/j.1600-0854.2007.00565.x)
Supplement: Supplementary Figures and Movies Legends [file EMS198606-supplement-Supplementary_Figures_and_Movies_Legends.pdf]

Supplementary Materials

Figure S1: Titration of effect of cholesterol depletion agents and actin drugs on uptake of fluid and Tf. FRαTb cells were treated with carrier or the drug at the concentrations indicated in duplicate dishes for 30 min, methyl-β-cyclodextrin; 15 min, filipin or 5 min (Lat A, Cyto D, Jas). After treatment, cells were assayed for their endocytic activity, fixed and imaged. Histograms represent fluid and normalized Tf uptake in cells, expressed relative to corresponding control cells (control values set to 1). Values represented are wt. mean ± SEM across two to three experiments.

Figure S2: Single-molecule imaging of GFP-tagged molecules. A) Characterization of single-molecule imaging: 1 nM solution of GFP (in PBS) was taken on acid-cleaned glass coverslips. GFP molecules, which settled down on coverslip, were imaged under TIRF illumination and high on-chip multiplication gain, leading to single-molecule sensitivity. Intensity traces of a single spot (montage) show that single molecules bleached in single step (Video S3). B) Bleaching profile of a single GFP molecule on coverslip. As has been described before, single GFP molecules show large intensity fluctuations, which are attributed to light-driven spectral fluctuations in the GFP chromophore (1). C) Histogram shows bleaching times of single molecules as a function of laser power applied, confirming that the disappearance of single molecules is a function of laser power and hence because of photobleaching (2). D) Cartoon shows how depth of TIRF field was calibrated. Dimensions of 1.2-μm bead were measured under wide field and evanescent field. Note we use objective-type TIRF; beads-cells are observed using an inverted microscope setup with the sample being illuminated from beneath. A decrease in diameter of fluorescent bead indicates, when illumination is changed from wide-field to TIR mode, change in depth of illumination. An estimation of the change gives a means to calculate the depth of TIRF field. E–F) Histograms show the comparison of distribution of fluorescence intensity from single molecules of GFP-Cdc42, single GFP molecules on coverslip or cytosolically expressed GFP (pEGFP-N1). Note that GFP-Cdc42 when expressed at fleetingly low levels appeared as punctate structures on the plasma membrane under TIRF illumination. Intensity distributions of these punctuate structures matched with those of eGFP molecules on coverslip. G) Cytosolically expressed eGFP diffuses in cytosol and it bounces off plasma membrane, residing for not more than 1–2 frames (∼100 ms). Intensity distribution of these events again matched with GFP-Cdc42, consistent with single molecules of GFP-Cdc42 being recruited to the plasma membrane. Moreover, cells expressing GFP-Cdc42 were fixed and molecules were bleached. Single molecules of GFP-Cdc42 bleached in a single step *in situ* (data not shown). H) Maximum projection from a video of GFP molecules expressed in single cell shows all the molecules that ever visited the TIRF field (membrane) in the 10 s. Notice that in absence of any membrane-specific localization, the number of molecules entering TIRF field are very limited (compare with Cdc42-WT or N17; Figure 6A,B; see also Video S3). Scale bar, 5 μm.

Figure S3: Actin and PH domain distribution in cholesterol-depleted cells. A) Images show cells transfected with PH domain-GFP before and after filipin treatment. PH domain redistributes to cytosol and nucleus and recovers back onto membrane after cholesterol addition. B) Distribution of polymerized actin in control cells or cells treated with 10 mM MβCD or 15 μg/ml filipin. Cells were depleted of cholesterol with either MβCD or filipin and stained with A488 phalloidin to mark F-actin. C–D) Dynamin2a-GFP-transfected cells were imaged live at high sensitivity before and after cholesterol depletion. Numbers in the images denote dynamin events recorded in that cell. Many of these events co-localize with A568-Tf (data not shown). Histogram shows the timescale of these events. Note that the number or timescale of residence of dynamin 2aa events does not change upon cholesterol depletion. Scale bar, 10 μm.

16000854, 2007, 6, Downloaded from https://onlinelibrary.wiley.com/doi/10.1111/j.1600-0854.2007.00565.x by University Of Warwick, Wiley Online Library on [08/09/2024]. See the Terms and Conditions (https://onlinelibrary.wiley.com/terms-and-conditions) on Wiley Online Library for rules of use; OA articles are governed by the applicable Creative Commons License

Video S1: Co-localization of GFP-Cdc42 with PLR-labelled FR-GPI: FRαTb cells transfected with GFP-Cdc42 and labelled with PLR at low concentrations (1/20th of the saturation level for this probe) were imaged at 37°C with dual colour TIR illumination (488 nm, and 543 nm laser lines). GFP-Cdc42 (green) and PLR (red) were recorded at 1.5 s per frame, video displayed at 2 fps to show dynamic regions at or near to the plasma membrane showing membrane regions enriched in both Cdc42 and FR-GPI. First frame shows the whole cell, subsequently a × 2.5 magnified area (square box) is shown at the same frame rate. Scale bar, 2 μm.

Video S2: Co-localization of GFP-Cdc42 with actin: FRαTb cells transfected with GFP-Cdc42 and Cherry-actin were imaged at 37°C with dual colour TIR illumination (488 nm, and 543 nm laser lines). GFP-Cdc42 (green) and Cherry-actin (red) were recorded at 1.5 s per frame, video displayed at 2 fps to show dynamic regions at or near to the plasma membrane showing membrane regions containing both Cdc42 and Cherry-actin. Scale bar, 2 μm.

Video S3: Single molecules of GFP on coverslip and expressed in cell cytosol: GFP molecules from a dilute solution were allowed to settle down on acid-cleaned glass coverslip or expressed in cytosol of FRαTb cells. Single molecules of GFP were imaged using TIR illumination; videos recorded at 12 fps and displayed at 2 fps. GFP molecules bleach in a single step, whereas the cytosolic GFP visits the membrane, appearing for a maximum time of one to two frames (from 10 to 90 ms). Scale bar, 2 μm.

Video S4: Video of single molecules of GFP-tagged isoforms of Cdc42-WT, N17, L61 and CRIB being recruited to membrane: FRαTb cells expressing different GFP-tagged proteins were imaged with TIR illumination (488 nm laser line) at 37°C. Single molecules of GFP-tagged Cdc42-WT, N17, L61 and CRIB are recruited onto membrane where they show diffusive (N17) or localized activity. Videos were recorded at 12 fps (GFP-CRIB at 2 fps) and are displayed at 2 fps. Scale bar, 2 μm.

Video S5: Effect of cholesterol depletion and repletion on GFP-Cdc42-WT dynamics at or near the plasma membrane: Cells expressing GFP-Cdc42 were imaged before and after cholesterol depletion and re-addition. Videos were recorded at 12 fps and displayed at 2 fps. Scale bar, 2 μm.

Video S6: Effect of cholesterol depletion on GTPase-deficient Cdc42L61: cells expressing GFP-Cdc42-L61 were imaged before and after cholesterol depletion. Videos were recorded at 12 fps and displayed at 2 fps. Scale bar, 2 μm.

Video S7: Effect of cholesterol depletion of actin–GFP dynamics: cells expressing GFP-Cdc42-L61 were imaged before and after cholesterol depletion and re-addition. Videos were recorded at 12 fps and displayed at 2 fps. Scale bar, 2 μm.

Video S8: Effect of cholesterol depletion on GFP-Rac1: cells expressing GFP-Rac1 were imaged before and after cholesterol depletion. Videos were recorded at 12 fps and displayed at 2 fps. Scale bar, 2 μm.

Video S9: Effect of cholesterol depletion on PH domain-GFP distribution: cells expressing PH domain GFP were imaged before and after cholesterol depletion. Videos were recorded at 12 fps and displayed at 2 fps. Scale bar, 2 μm.

Video S10: Effect of cholesterol depletion on GFP-dynamin 2aa: cells expressing GFP-dynamin 2aa were imaged before and after cholesterol depletion. Videos were recorded at 0.5 fps and displayed at 2 fps. Scale bar, 2 μm. Note: The videos must be viewed with relatively low-ambient lighting because the contrast of individual frames has been kept constant throughout the video sequence.

Supplemental materials are available as part of the online article at http://www.blackwell-synergy.com

16000854, 2007, 6, Downloaded from https://onlinelibrary.wiley.com/doi/10.1111/j.1600-0854.2007.00565.x by University Of Warwick, Wiley Online Library on [08/09/2024]. See the Terms and Conditions (https://onlinelibrary.wiley.com/terms-and-conditions) on Wiley Online Library for rules of use; OA articles are governed by the applicable Creative Commons License
